# LETTER TO THE EDITOR Reply to: Anatomical Landmarks for Safe Elevation of the Deep Inferior Epigastric Perforator Flap: A Cadaveric Study by Chowdhry *et al*

**Published:** 2012-08-23

**Authors:** Kwok Hao Lie, Warren Matthew Rozen, Iain Stuart Whitaker, Mark Winter Ashton

**Affiliations:** Taylor Laboratory, University of Melbourne and Royal Melbourne Hospital

Dear Sir,

We read with great interest Chowdhry et al's article about anatomical surface landmarks for predicting perforator location prior to deep inferior epigastric artery perforator flap raising.

Although the ability to predict the approximate location of a perforator based upon surface anatomical landmarks should reduce the amount of time required for preoperative Doppler assessment of perforator location, many surgeons have ceased the use of such ultrasound-based perforator localization due to discrepancies between the ultrasound and intraoperative findings,^1^ as well as the proven superiority of computed tomographic angiography over Doppler ultrasound in visualizing perforator anatomy.^2^

As the authors have written in their article, computed tomographic angiography (CTA) and magnetic resonance angiography (MRA) allow the surgeon to visualize the arterial anatomy of the entire anterior abdominal wall in advance of the actual operation. Three dimensional reconstructions of the abdominal wall from such investigations allow for “virtual dissection” of the perforators, providing accurate and comprehensive information regarding the origin, intramuscular course, and anterior rectus sheath exit point of any perforator of clinical interest, which is more than 0.3 mm in diameter.^3^

With such detailed anatomical information available, the previous “fear of the unknown” when approaching perforator dissection without preoperative perforator imaging has been overcome. This has been reported to reduce operative times^4^ and conversions to alternative surgical techniques such as muscle sparing free transverse rectus abdominis myocutaneous flaps.^5^ The reduction in operative times also translates to significant reductions in health care costs, which more than covers the cost of the initial preoperative investigation.^6^ Such are the benefits of CTA and/or MRA that many large reconstructive centers now perform it routinely in all their patients undergoing DIEAP (deep inferior epigastric artery perforator) flap reconstructions.^7^

Therefore, in the context of planning DIEAP flaps, we strongly advocate the use of CTA and/or MRA as local resources and expertise permit, which would in itself remove any preoperative uncertainty about the vascular anatomy that the surgeon will find during dissection.
